# Leg length and offset in short-stem total hip arthroplasty: is a single offset-implant sufficient to restore the hip rotation centre within a range of 5 mm?

**DOI:** 10.1007/s00402-026-06194-7

**Published:** 2026-02-02

**Authors:** Felix Olk, Bernd Bittersohl, Jürgen Babisch, Hagen Mittelstädt, Marcus Jäger, Rüdiger Krauspe, Christoph Zilkens

**Affiliations:** 1 Department of Orthopaedic Surgery Ex-Director: Univ.-Prof. Dr. med. R. Krauspe, Heinrich Heine University Düsseldorf, Düsseldorf, Germany; 2 Department of Trauma Surgery, Orthopaedics and Hand Surgery, Rheinland Klinikum Neuss GmbH, Neuss, Germany; 3 Department of Orthopaedic Surgery Director: Univ.-Prof. Dr.med. B. Bittersohl Klinikum Bielefeld gem. GmbH, Bielefeld University, Bielefeld, Germany; 4 Department of trauma, hand and orthopaedic surgery, Helios Klinikum Erfurt, Erfurt, Germany; 5 Department of trauma and orthopaedic surgery, University Hospital Schleswig-Holstein, Lübeck, Germany; 6 Department of Orthopaedic Surgery Director: Univ.-Prof. Dr. med. M. Jäger St.Marien-Hospital Muelheim an der Ruhr GmbH, University of Duisburg-Essen, Essen, Germany; 7 MVZ OGPaedicum GmbH, Bochum, Germany

**Keywords:** Total Hip Arthroplasty, Femoral Offset, Leg Length, Templating, Short stem, CCD Angle, Caput-Collum-Diaphyseal Angle, Extended Offset, Varus, Calcar-Guided

## Abstract

**Introduction:**

Short-stem total hip arthroplasty (THA) has gained popularity due to its bone-preserving properties and improved physiological load transmission to the proximal femur. Despite these advantages, the ability of short-stem implants to reliably restore leg length and offset remains debated. This study evaluates whether a single offset implant is sufficient for accurate anatomical reconstruction or if multiple offset options are necessary.

**Material and methods:**

A total of 148 anteroposterior pelvic radiographs of patients scheduled for short-stem THA were analysed using MediCAD^®^ software. Femoral offset and leg length were measured, and the accuracy of anatomical reconstruction was assessed within a 5-mm target range. Comparisons were made between a single offset (130°) implant and a dual-offset system (130° and 119°) using the McNemar-Bowker test.

**Results:**

With a single 130° CCD (Caput-collum-diaphyseal) offset implant, 55.7% (82/148) of cases achieved satisfactory leg length and offset restoration. The use of a dual-offset system improved accuracy to 79.1% (117/148), demonstrating a statistically significant advantage (*p* < 0.001).

**Discussion:**

The study highlights the need for at least two CCD-angle-offset combinations in short-stem THA to address anatomical variability. A dual-offset system enhances accuracy, reduces biomechanical imbalances, and therefore we expect improvements in clinical outcomes, particularly in teaching hospitals where standardization is essential.

## Introduction

Short stem total hip arthroplasty (THA) has gained popularity as an alternative to conventional THA due to its advantages in bone preservation, improved physiological load transmission through the proximal femur, and the ability to reconstruct coronal hip anatomy, including the anterior femoral offset [1–3]. Long-term studies have demonstrated excellent survival rates and clinical outcomes for short-stem THA [1, 4, 5]. In Germany, the proportion of short stem implants in hip arthroplasty has steadily increased, reaching 15.1% in 2023 [6].

Short stem THA is primarily designed for metaphyseal anchorage [7], but different implant systems vary in their bone resection approach and anchoring can be individualized [8].

These implant systems include femoral neck-preserving systems, which retain most or all of the femoral neck; femoral neck-compromising systems, which remove part of the femoral neck; and femoral neck-resecting systems, which remove the femoral neck entirely. However, optimal implant choice remains a topic of debate since some short stem designs have limitations in accurately reconstructing leg length and femoral offset [9].

Femoral offset and leg length are critical parameters for restoring joint biomechanics and stability. Achieving anatomical reconstruction is crucial as it significantly influences gait function, long-term stability, and overall patient satisfaction.

Imprecise reconstruction might cause gait disturbances, leg length discrepancies and alterations in the abductor moment arm, often followed by increased wear or the risk of dislocation [10].

These parameters depend on the centre of rotation of the femoral head, defined by horizontal and vertical offsets, and the stem’s position within the proximal femur [11].

The combination of the offset and Caput-Collum-diaphysis-angle (CCD-angle) provides a reference for understanding the relationship between these factors. Calcar-guided short stem THA is intended to enable precise restoration of joint geometry while preserving bone. However, certain designs require specific varus or valgus positioning for optimal reconstruction, as described in the “top-down concept” [1]. This concept sets the rotation centre of the cup at the “top”, then a stem with a “fit-and-fill” in the femoral flare is inserted. The femoral cut level of the neck is set by the position of the stem, which is determined by the individual hip anatomy. A valgus hip deformity is therefore reconstructed with a more distal resection level and a straighter stem positioning, while a varus hip is reconstructed with a more proximal cut level and an increased femoral offset.

Clinical data indicate that the level of femoral neck resection significantly influences the post-operative CCD angle and offset [12]. A more inferior resection level tends to induce valgus positioning, reducing the offset and potentially leading to gluteal insufficiency. In such cases, the options for correction include using a greater offset stem or adjusting the resection level.

Jerosch [13] suggested that anatomical reconstruction of hip geometry can be achieved with a single-offset implant by modifying the femoral neck resection level. However, this approach highly depends on surgical experience and can lead to inconsistent outcomes. In high-offset cases, precise reconstruction using a single CCD-angle implant often necessitates a near-subcapital resection, which requires experience and meticulous pre-operative templating. However, the long-term stability of highly varus-positioned stems remains uncertain [12], despite promising overall outcomes [13].

Given the growing use of short-stem THA in teaching hospitals, standardization of surgical techniques is essential for achieving reproducible results, even among less experienced surgeons.

Various short-stem designs have been developed to address these challenges, differing in femoral resection levels and anchoring techniques [14].

To minimise variability in neck resection and implant positioning, this study assesses whether leg length and femoral offset can be reliably reconstructed using a single CCD-angle-offset combination in short stem THA. Alternatively, we seek to determine whether a second CCD-angle-offset combination is necessary to improve reconstruction accuracy.

## Materials and methods

### Patient selection

**Fig. 1:**
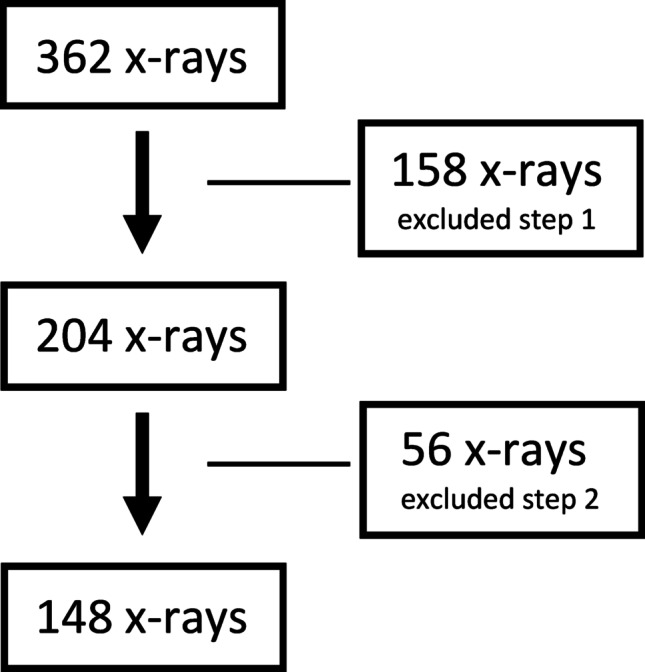
Patient flow chart: 362 pre-operative supine anteroposterior (AP) pelvic X-rays collected from five centres from patients who underwent short stem total hip arthroplasty (THA) were analysed regarding image accuracy. The remaining 204 X-rays were further assessed for symmetry and orientation

This study analysed 362 pre-operative plain radiographs collected from five centres to assess their suitability for inclusion. All radiographs were obtained from patients who underwent short stem total hip arthroplasty (THA) and were taken as supine anteroposterior (AP) pelvic X-rays, following standard recommendations (Fig. 1).

In the first selection step, 158 radiographs were excluded based on the following criteria:

1. Severe dysplasia (Tönnis III or Crowe ≥ II) (*n* = 62), an existing or scheduled implant on the contralateral side (*n* = 7), which could impair the precise determination of the unaffected hip’s rotation centre.
2. Lack of a calibration sphere (25 mm), preventing accurate scaling (*n* = 10).
3. Poor image quality caused by obesity, incorrect X-ray intensity, or blurriness (*n* = 23).
4. Extreme pelvic tilt, with > 50% size difference between the sides, measured by the surface content of the obturator foramen (*n* = 33).
5. Severe external leg rotation, identified by a > 50% width difference between both lesser trochanters, which affects the accurate measurement of the CCD angle and femoral offset (*n* = 8).
6. Covered anatomical landmarks (e.g., teardrop or ischial tuberosity) due to gonadal shielding (*n* = 15).

In the second step, 204 remaining radiographs of patients awaiting unilateral hip replacement with an indication for a short stem arthroplasty were further assessed for symmetry and orientation, following the criteria of Tannast et al. 2007 [15] and Amenabar et al. 2015 [16]. An additional 56 radiographs were excluded due to:

7. Pelvic rotation, defined as a > 1 cm deviation between the centre of the symphysis and sacrococcygeal joint (*n* = 11).
8. Pelvic tilt, assessed by the vertical distance between the upper border of the symphysis and the sacrococcygeal joint (*n* = 6).
9. Significant contralateral femoral offset differences (> 8 mm, *n* = 39).

Based on the neck-shaft-angle (CCD angle), patients were categorized into three groups:

10. Group A (Varus): CCD angle < 125° (47%).
11. Group B (Neutral): CCD angle 125°–130° (33%).
12. Group C (Valgus): CCD angle > 130° (20%).

**Fig. 2:**
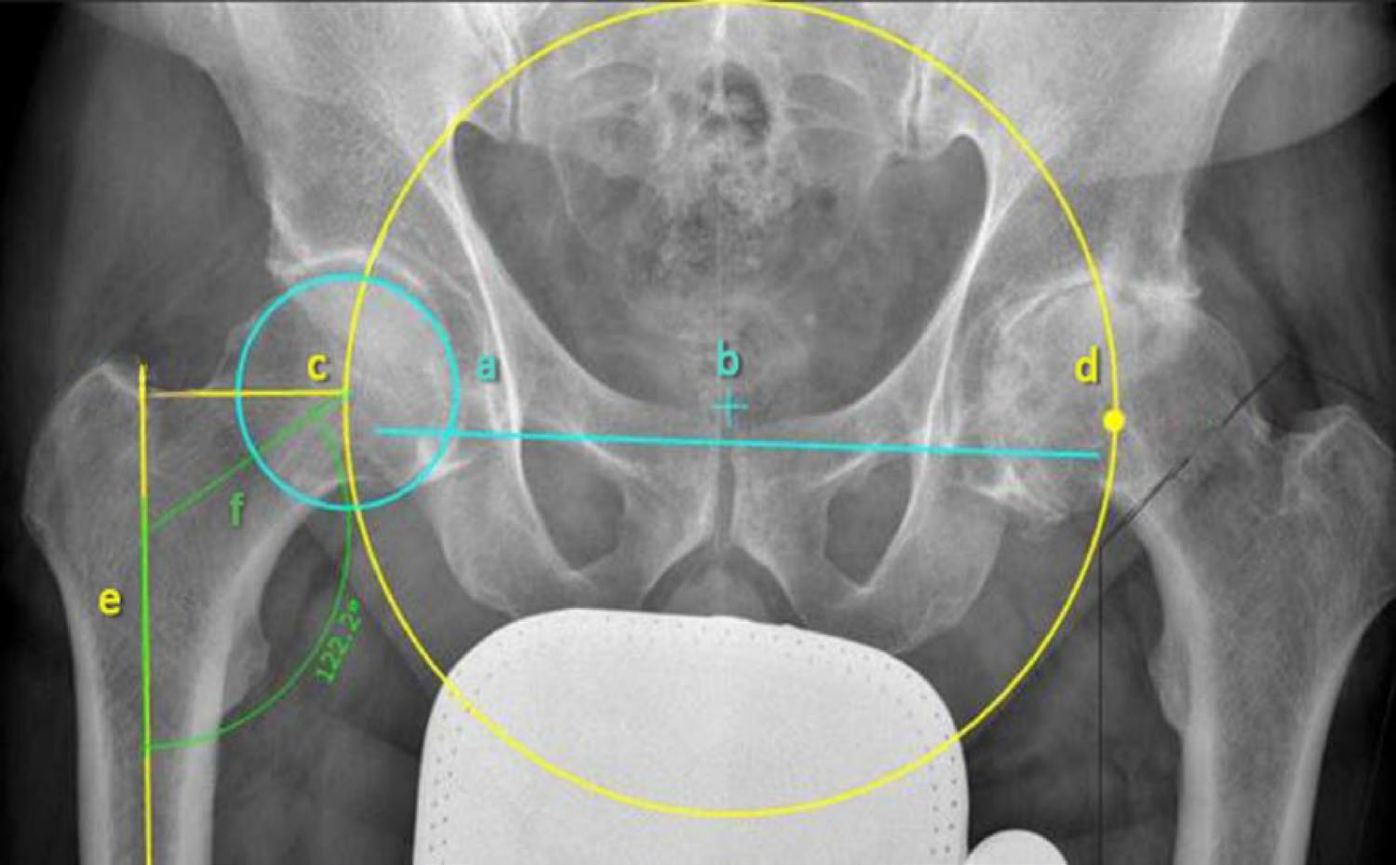
Digital templating of the cup and stem position. The circle’s centre was positioned at the intersection of the horizontal pelvic axis (**a**) and the vertical symphysis axis (**b**). The centre of femoral rotation (**c**) on the unaffected side was mirrored to the affected side using a circular reference. A parallel line to the horizontal hip axis was used to determine the mirrored rotation centre (**d**). The stem was planned with a maximum of 10° varus or valgus positioning relative to the femoral axis (**e**). The CCD angle (122,2°) of the unaffected side was measured between the femur axis (**e**) and the axis of the femoral neck (**f**)”

**Fig. 3:**
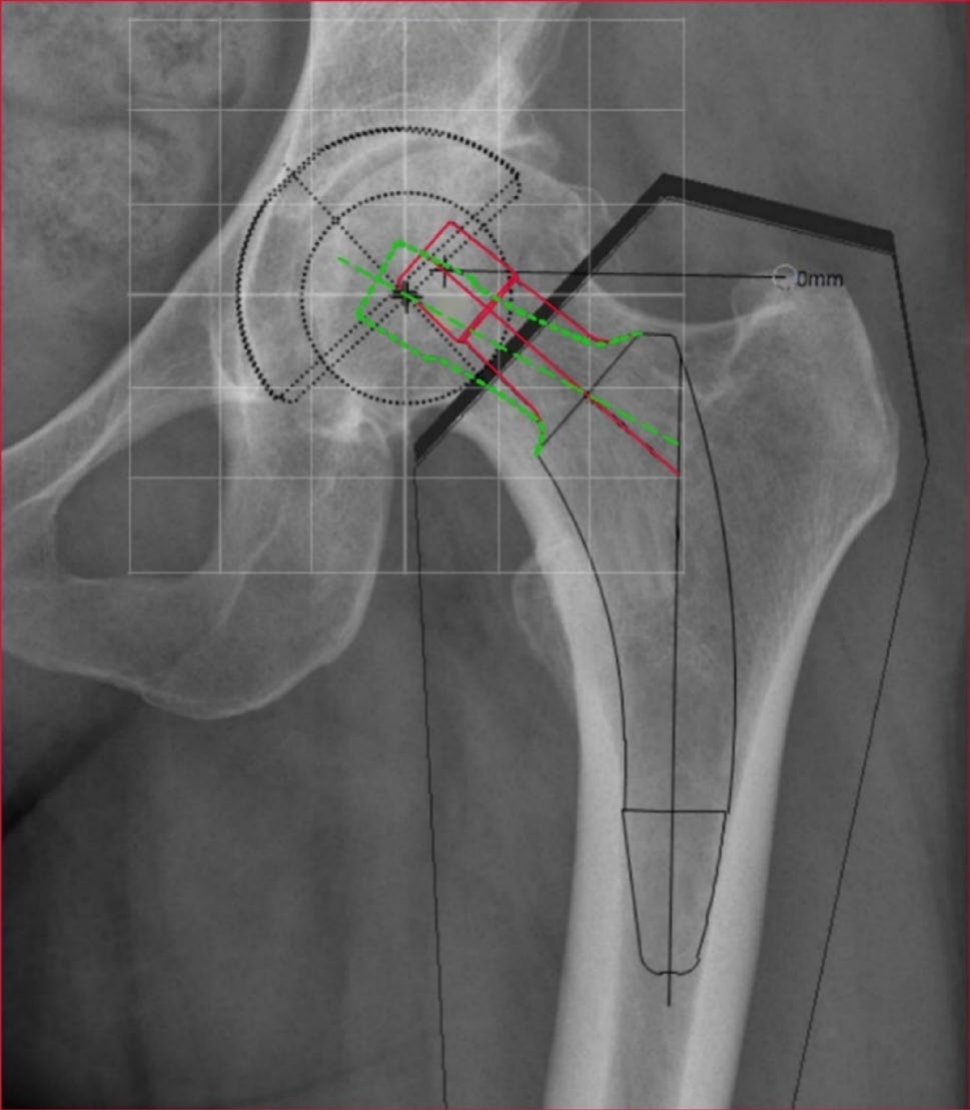
The red continuous template represents the current stem design with an 130° and the green-dashed template the new 119° CCD-angle. The square diagram is not original size and serves only for explanation of the cloud diagram in Fig. 5

All THA were pre-operatively planned by one reviewer (fo) using the MiniHip template (Corin SA, UK) with MediCAD^®^ software (Hectec GmbH, Aldorf bei Landshut, Germany) used for digital templating of the cup and stem position, following manufacturer guidelines [17] (Fig. 2). Templating and measurements were carried out by one reviewer (FO) and were matched with a sample (*n* = 50), which were carried out by a second reviewer (CZ). Intraobserver and interobserver reliabilities were calculated using correlation coefficients for the average measurement and a Two-way mixed effects model for the absolute agreement definition. Therefore, the centre of femoral rotation on the unaffected side was mirrored to the affected side using a circular reference. The circle’s centre was positioned at the intersection of the horizontal pelvic axis (a) and the vertical symphysis axis (Fig. 3). A parallel line to the horizontal hip axis was used to determine the mirrored rotation centre (d). Leg length adjustment was performed by aligning the lesser trochanter (Tr. minor) of the operated side to the height of the unaffected side, using the horizontal pelvic axis (a) as a reference. The acetabular cup was planned at a 45° inclination relative to the horizontal pelvic axis (a). The largest fitting stem size was inserted with a caudal press-fit into the substantia compacta of the diaphysis, ensuring alignment with the cortical bone line. A medium head size was used consistently to standardize comparisons across cases. To provide standardized implant positioning, the calcar-guided stem was planned by using a proximal “fit and fill” technique, with a maximum of 10° varus or valgus positioning relative to the femoral axis (e).

### Data collection and statistical analysis

Microsoft Excel 16.0 (Redmond, USA) was used for data collection and visualization in pivot tables. Radiograph rotation was considered by stringent selection criteria regarding the femoral and pelvic sagittal rotation as well as the horizontal pelvic tilt.

Leg length and femoral offset differences were calculated as follows:

13. Femoral offset difference: measured as the horizontal distance between the anatomical and artificial centre of rotation from the template.
14. Leg length difference: measured as the vertical distance between the two centres.

A 5-mm target window was set as the threshold for accurate implant positioning to minimise deviations between the stem and cup centres. The distribution of leg length and offset differences for the 148 selected cases was visualized in a coordinate system (Figs. 3 and 5).

An iterative calculation was performed to determine whether a single CCD-angle-offset combination was sufficient for reconstruction. Different CCD angles were tested to maximize the number of cases within the 5-mm target range. The best results were achieved using a combination of:

15. A standard 130° stem.
16. A lateralised 119° stem as an alternative.

Both stem designs were simulated for each case (Fig. 3), and the more accurate stem type was selected for final analysis. The optimized 148 rotation centres were then visualized and categorized by stem type (Fig. 5).

The SPSS 24 statistical software package (IBM Corp., Armonk, NY, USA) was used for statistical evaluation. Demographic variables (e.g., sex, age, CCD angle) were analysed before and after selection. Implant accuracy was analysed within 5-mm, 3-mm, and 1-mm target ranges. Categorical variables were compared using Fisher’s exact test or the Chi-square test. Continuous variables were assessed for normal distribution and analysed using the student’s t-test. Paired nominal data were analysed applying the McNemar-Bowker test. A p-value of ≤ 0.05 was considered statistically significant for all tests.

## Results

The interobserver and intraobserver correlation coefficients were good to excellent (range, 0,82 [95% CI 0,73 − 0,89] to 0,99 [95% CI 0,98 − 0,99]) with good agreements for horizontal positioning of the stem and stem inclination. Excellent agreements were found for the choice of implant size and vertical positioning, both for the stem and the cup.

The patient cohort exhibited a normal Gaussian distribution of anatomical CCD angles in terms of symmetry and profile, with a mean of 125.40°± 5.32 (range: 114° – 139°) tendency trend toward lower CCD angles with increasing age was observed.

The 148 cases were categorized into three CCD-angle groups:

17. Varus Group (A): 69 cases (< 125°).
18. Neutral Group (B): 49 cases (125°–130°).
19. Valgus Group (C): 30 cases (> 130°).

The differences in leg length and femoral offset for each group are summarized in Table 1. A higher offset difference (– 3.8 mm) and increased leg length (+ 1.9 mm) were noted within the varus group. The valgus group revealed a lower offset difference (0.10 mm) and a negative leg length difference of – 1 mm. The leg length difference for the valgus group C was negative (– 1 mm).

**Table 1:** Offset and leg length differences

Group	Mean	Std. deviation	N
Offset difference in cm			
A: varus (CCD: <125)	− 3.81	4.20	69
B: neutral (CCD: 125–130)	− 2.49	4.18	49
C: valgus (CCD: >130)	0.10	4.10	30
Total	− 2.58	4.40	148
Leg length difference in cm			
A: varus (CCD: <125)	1.94	3.52	69
B: neutral (CCD: 125–130)	− 0.04	3.92	49
C: valgus (CCD: >130)	− 1.00	4.62	30
Total	0.69	49.06	148

**Fig. 4:**
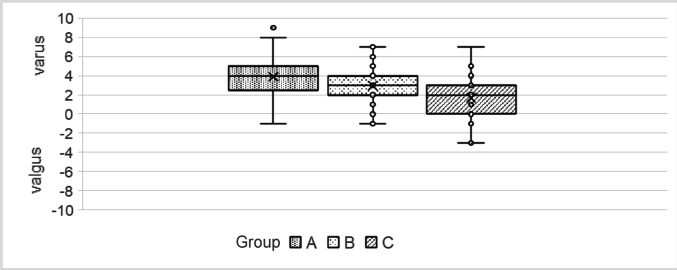
Distribution of the angular deviation of the technical stem axis towards the femoral axis between CCD-groups

All stems were planned in a slight varus-oriented position, with an average of 3° varus (Fig. 4):

20. Varus Group (A): 4°.
21. Neutral Group (B): 3°.
22. Valgus Group (C): 1.8°.

Due to this orientation, the femoral offset was slightly larger than the artificial offset of the stem, particularly in the varus group compared to the valgus group.

**Fig. 5:**
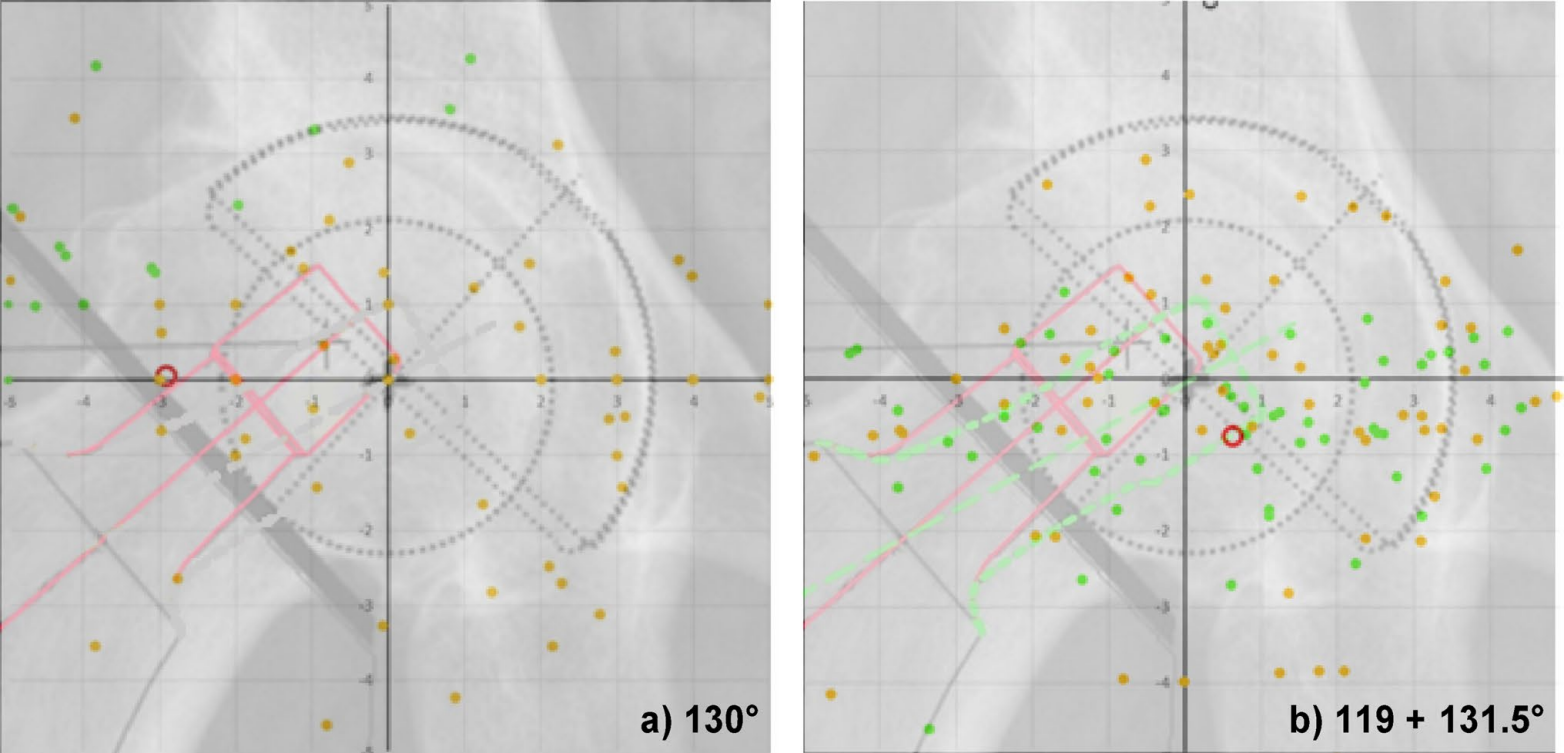
Cloud diagrams showing the reproduction of the centre of rotation inside the 5 mm range. Diagram **a** All cases were planned using a 130° stem angle. The green circles indicate cases that would profit from a 119° stem-angle. **b** All cases were planned using a 119° (green) or 131.5° (orange) stem angle. The distribution of the circles is more medialized and closer to the x-axis, resulting in less leg-length and offset difference for these cases

Using a two-stem CCD design (131.5° and 119°), 117 cases (79.8%) were within the target range. This resulted in a statistically significant increase of 35 cases (+ 23.4%) (*p* < 0.001, McNemar-Bowker Test). Significant improvements were observed in Groups A and B (Table 2). In contrast, no significant difference was found for leg length between cup-adjusted and two-stem design templating across all three groups (*p* = 1.000). For the varus group (A), the two-stem approach increased cases within the 5 mm target window by 27 cases (+ 18.24%) Fig. 5.

**Table 2:**
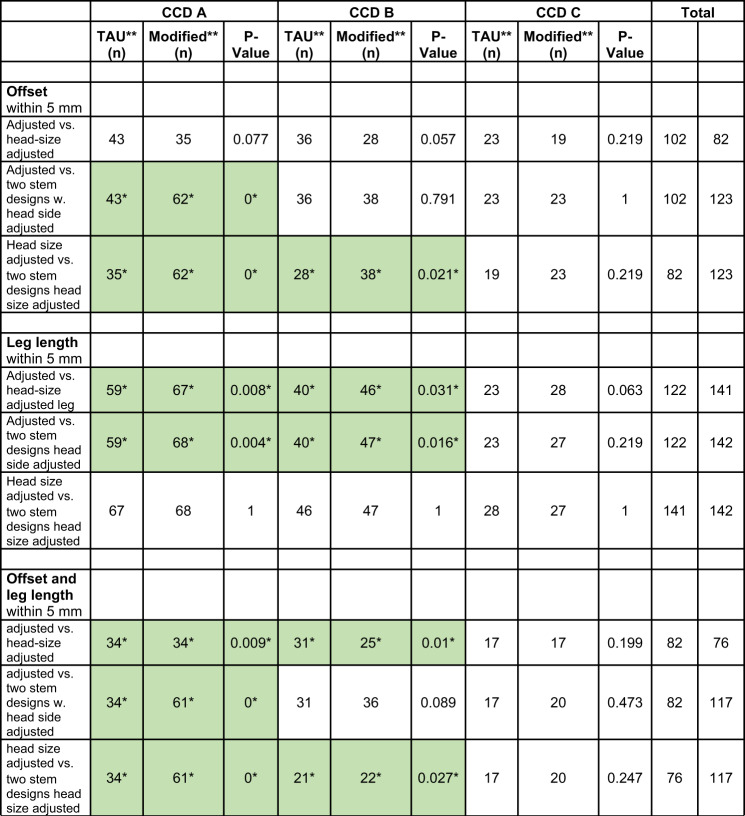
Outcomes inside 5 mm target range for leg length and offset compared by McNemar Chi-Square test for each CCD-group

**TAU = first variable in each table row, Modified = second variable

*p < 0.05 (green-coloured)

## Discussion

This study explored two key hypotheses: (1) Can a single CCD-angle implant adequately reconstruct leg length and femoral offset in short-stem THA? (2) Would an additional CCD-angle option improve reconstruction accuracy? Using standardized pre-operative planning with a maximum stem inclination of 10°, we assessed whether a single CCD-angle stem can reliably restore hip anatomy. The study cohort was comparable in age, sex, and CCD-angle distribution to previous short-stem studies [18], with no statistical bias or distortion in cohort selection.

In this study, higher offset and leg length discrepancies (Table 1) suggest the limitations of a fixed CCD-angle approach, particularly in varus (Group A) and neutral (Group B) hips. A significant improvement in reconstruction was observed when an additional CCD-angle option was introduced, especially in Groups A and B. Valgus hips (Group C) exhibited slight negative leg length discrepancies, likely due to varus-oriented positioning at 10°. A more varus positioning could have further minimised this discrepancy. The commonly used ± 5 mm target range showed significant improvements with a second CCD-angle option, supporting its clinical utility [9, 19]. Within the ± 3 mm range, statistical analysis indicated better restoration of the anatomical rotation centre, demonstrating the superior precision of the dual-stem approach. Vorimore et al. [20] reported that a ± 2.5 mm range can be achieved in only 10% of the cases. No significant differences were observed in the ± 1 mm range, though some cases still benefited from a second CCD-angle. While no prior studies have explored a 1 mm reconstruction target in short-stem THA, advancements in CT-based 3D templating and guided surgical techniques may make this achievable [21].

Our results align with and differ from previous studies. Similar to earlier research on short-stem CCD angles, no significant differences were found between male and female patients regarding leg length and offset restoration [3]. Wedemeyer et al. [22] reported strong correlations between templated and intra-operatively used sizes (MAYO^®^, Zimmer) but poor correlation with CCD angle, leg length, and offset. Schmidutz et al. [23] demonstrated high pre-operative planning accuracy using the Metha^®^ stem, with no significant difference in templating accuracy between short-stem and conventional THA. Höhle et al. [24] compared pre-operative templating to post-operative outcomes for Metha and MAYO short stems, finding a post-operative femoroacetabular offset loss and larger-than-expected leg length. They also noted a valgus shift in stem positioning, underscoring the intra-operative difficulty of achieving planned positioning. This offset change was attributed to stem design, leading to a demand for a lateralised stem (offset plus). While previous studies suggested an additional offset option, our findings support the need for a second CCD-angle stem, simplifying pre-operative planning by identifying cases benefiting from a second stem type through CCD-angle measurement.

Other prosthetic designs, such as the Collo-MIS, are based on anatomical shapes [25–27], while implants like the Mini-Hip (Corin), Fitmore (Zimmer), and Nanos (Smith & Nephew) were developed using CT databases. The Optimys stem has been analysed post-operatively with mediCAD software, showing minimal offset increases and high leg length reconstruction rates [28]. Although Jerosch et al. [3] suggested that a single CCD-angle stem design fits femurs with CCD angles between 114° and 146°, this study indicates that a lateralised stem design is superior for varus hips with CCD angles below 125°. Girard et al. recently proposed a range of 120° to 150° for short stems [29]. However, as both previous studies and our findings indicate, the mean CCD angle is 125°, meaning many cases fall outside the accessible range suggested by Girard [29].

Erivan et al. [30] reported a more moderate femoral offset increase in short stems compared to standard stems but advocated for technique modifications to optimize clinical outcomes. A superior hip reconstruction for a 12 options short-stem design reconstructed femoral offset compared to a 24 options short-stem and a straight-stem with 76 options was reported by Maurer-Ertl et [7]. The acetabular offset decreased in all three groups, which could be explained by a stem independent moderate drilling into the acetabular subchondral sclerosis.

Brinckmann et al. [31] examined two short-stem types in terms of varus/valgus positioning and its impact on bone mineral density (BMD). While the offset and centre of rotation (COR) remained unchanged pre- and post-operatively, BMD changes were attributed to moderate stress shielding and more proximal load transfer. Kutzner et al. [32] observed initial subsidence due to trabecular metaphyseal bone integration, followed by gradual stabilization. Von Engelhard et al. [1] reported excellent long-term results for the Mini-Hip, with a 97.32% revision-free survival rate over at least nine years, making it a viable alternative to standard implants. Given the promising long-term outcomes of short-stem THA (MiniHip, Optimys, Fitmore, and Nanos) with survivorship rates of ~ 97% [1, 28], we anticipate even better results with a two-stem approach. Nevertheless, an individualized resection level remains essential for optimal reconstruction, with surgical expertise playing a critical role in post-operative accuracy.

While increased post-operative offset has been associated with improved range of motion, lower wear rates, and enhanced muscle strength [33], excessive offset may cause trochanteric pain and increased medial bending stress [33]. Previous studies suggest that a greater offset improves abductor strength and range of motion but does not significantly impact post-operative pain scores [34].

This study has several limitations: (1) reliance on pre-operative radiographs without post-operative confirmation, (2) planning conducted by only one surgeon, which may limit generalizability, and (3) the use of 2D radiographs instead of 3D imaging for assessing complex anatomical parameters. Like Innmann et al. [11], we compared the templating to a second reviewer to emphasize the significance and expect a slight FO underestimation, while the ratios in- and outside the target range should remain.

Nonetheless, radiographic templating remains the most accessible, cost-effective, and widely used method for THA planning [16].

Within the cohort, no cases of Dorr type c were found. We assume those cases have already been ruled out as unsuitable for cementless short stem anchoring [8], because a stove-pipe design increases the intramedullary freedom for stem positioning, while there is an inherent risk of early loosening, stem subsidence and intraoperative femur fractures [35].

## Conclusion

This study demonstrates the necessity of incorporating at least two CCD-angle options in short-stem THA to better accommodate anatomical variability. A dual-stem approach enhances pre-operative planning, improves intra-operative flexibility, and optimizes implant positioning while maintaining economic efficiency. Future research should focus on post-operative validation, 3D templating techniques, and long-term functional outcomes to refine short-stem THA strategies further.

## Data Availability

No datasets were generated or analysed during the current study.
